# Development and Validation of a Specific Stability Indicating High Performance Liquid Chromatographic Method for Valsartan

**DOI:** 10.4103/0975-1483.63166

**Published:** 2010

**Authors:** KS Rao, N Jena, MEB Rao

**Affiliations:** *Roland Institute of Pharmaceutical Sciences, Berhampur-760010, Orissa, India*

**Keywords:** Assay, stress studies, validation, valsartan

## Abstract

A stability-indicating HPLC assay method has been developed and validated for valsartan in bulk drug and pharmaceutical dosage forms. An isocratic RP-HPLC was achieved on Waters 2695 using Symmetry C18 (250mm × 4.6mm × 5μ) column with the mobile phase consisting of 0.02 mM sodium dihydrogen *ortho*-phosphate, pH adjusted to 2.5 using *ortho*-phosphoric acid (solvent A), and acetonitrile (solvent B) in the ratio of 58:42 %v/v. The stress testing of valsartan was carried out under acidic, alkaline, oxidative, thermal, and photolytic conditions. Valsartan was well resolved from its degradation products. The proposed method was validated as per ICH guidelines. The method was found to be suitable for the quality control of valsartan in bulk and pharmaceutical dosage forms as well as the stability-indicating studies.

## INTRODUCTION

**Figure 1:**
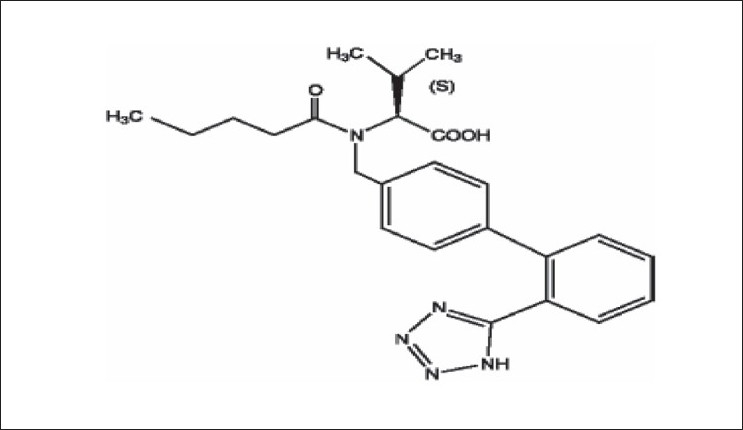
Chemical structure of valsartan

Valsartan is chemically described as *N*-(1-oxopentyl)-*N*-[[2’-(1H-tetrazol-5-yl) 1,1’-biphenyl]-4-yl]methyl]-L-valine [Figure 1]. Valsartan is a nonpeptide, orally active, and specific angiotensin II antagonist acting on the AT_1_ receptor subtype. Its empirical formula is C_24_H_29_N_5_O_3_, its molecular weight is 435.5. Valsartan is a white fine powder. It is soluble in ethanol and methanol and slightly soluble in water. It is available as tablets for oral administration, containing 40 mg, 80 mg, 160 mg, or 320 mg of valsartan.

According to current good manufacturing practices, all drugs must be tested with a stability-indicating assay method before release. Stress testing of the drug substance can help identify the likely degradation products, which can in turn help establish the degradation pathways and the intrinsic stability of the molecule and validate the stability-indicating power of the analytical procedures used. The nature of the stress testing will depend on the individual drug substance and the type of drug product involved. Keeping into the view of susceptibility of valsartan under variety of conditions, it was felt that a HPLC method of analysis that separates the drug from the degradation products formed under ICH suggested conditions (hydrolysis, oxidation, photolysis, and thermal stress)[1] would be of general interest. These studies provide valuable information on drug’s inherent stability and help in the validation of analytical methods to be used in stability studies.[2] A survey on literature reveals that a very few methods were developed for the estimation of valsartan in biological fluids and marketed formulations.[3–10] The literature has demonstrated that a stability-indicating LC method for determination of valsartan in the presence of its impurities and degradation products generated from forced decomposition studies was developed.[11] Nevertheless, in that reported study, the retention time of valsartan was obtained at 27 min. However, in our present method the retention time of valsartan was achieved at 9.3 min. Thus, the aim of our study was to develop a simple, selective, economic, specific stability indicating the LC method that can be used to determine the related substances and also the assay of bulk samples of valsartan. This paper deals with the development of stability indicating the analytical method using the samples generated from forced degradation studies.

## EXPERIMENTAL DETAILS

### Materials

Gift sample of valsartan was received from analytical research and development laboratory of International Speciality Product, Hyderabad, India. HPLC grade acetonitrile was purchased from Fischer Scientific, India. Analytical reagent grade sodium dihydrogen *ortho*-phosphate was purchased from S.D. Fine Chem. Limited, India, and hydrogen peroxide were purchased from Qualigens Fine Chemicals, India. High pure water was prepared by using Millipore Milli Q plus purification system. Valzar^®^commercial formulations were purchased from the local market.

### Instrumentation

The LC system, used for method development, forced degradation studies, and method validation was Waters 2695 series (manufactured by Waters Technologies, USA) LC system with a diode array detector. The output signal was monitored and processed using Empower software (designed by Waters Technologies, USA) on lenovo computer (Digital Equipment Co).

### Chromatographic conditions

The chromatographic column used was Waters using Symmetry C18 (250mm × 4.6mm × 5μ). The mobile phase consists of a mixture of 0.02 mM sodium dihydrogen *ortho*-phosphate, pH adjusted to 2.5 using *ortho*-phosphoric acid (solvent A), and acetonitrile (solvent B) in 58: 42 ratio. The mobile phase was pumped from the solvent reservoir to the column at a flow rate of 1 ml/min for 20 min. The column temperature was maintained at 23 ± 1 °C. The eluate was monitored at 250 nm using the PDA detector. The injection volume was 10 (μl). Mobile phase was used as diluent during the standard and test samples preparation.

### Preparation of standard solutions

Stock solution of valsartan was prepared by dissolving 25 mg of valsartan in 25 ml of volumetric flask containing 20 ml acetonitrile. The solution was sonicated for about 20 min and then made up to volume with acetonitrile. Aliquots of the standard stock solution of valsartan were transferred using A-grade bulb pipettes into 10 ml volumetric flasks and the solutions were made up to volume with mobile phase to give final concentrations of 1,5,50,100,150, and 200 μg/ml.

### Preparation of valsartan tablets for assay

Twenty tablets were weighed, finely powdered, and an accurately weighed sample of powdered tablets equivalent to 25 mg of valsartan was treated with acetonitrile and phosphate buffer (pH 2.5) in a 25 ml volumetric flask using ultra sonicator. This solution was filtered through 0.45 μm filter paper. Suitable aliquot of the filtered solution was added to a volumetric flask and made up to volume with mobile phase to yield a starting concentration of 0.1 mg/ml.

### Forced degradation studies

In order to determine whether the analytical method and assay were stability-indicating, valsartan tablets and valsartan active pharmaceutical ingredient (API) powder were stressed under various conditions to conduct forced degradation studies. Intentional degradation was attempted to stress conditions acid hydrolysis (using 0.75N, 1.5N HCl), base hydrolysis (using 1.5N NaOH), oxidative degradation (using 3.0%, 10% H_2_O_2_), photolytic degradation (UV cabinet, an overall illumination of ≥210 Wh/m^2^ at room temperature with UV radiation at 320–400 nm), and thermal treatment (heated at 50°C, 80°C for 2 h) to evaluate the ability of the proposed method to separate valsartan from its degradation products. Valsartan at a concentration of 0.5 mg/ml was used in all the degradation studies. After completion of the degradation processes, the solutions were neutralized and diluted with mobile phase.

### Acid and alkaline degradation

**Figure 2:**
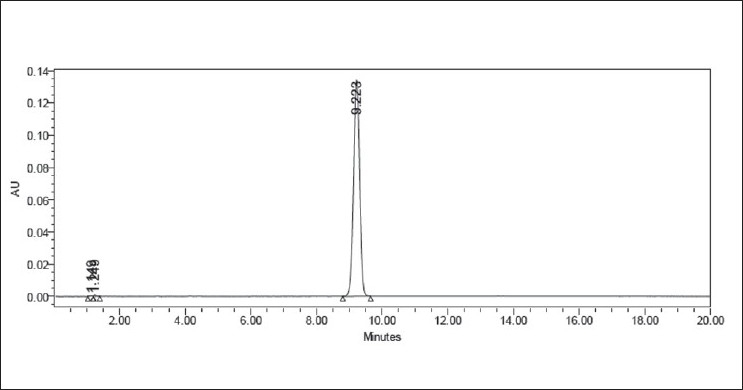
Typical chromatogram representing the degradation behavior of valsartan in acid hydrolysis

**Figure 3:**
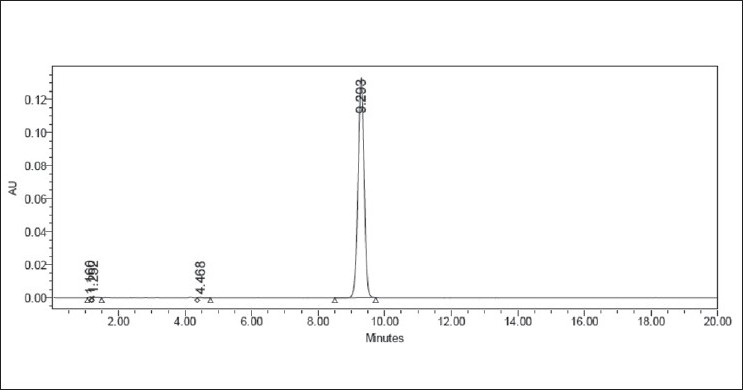
Typical chromatogram representing the degradation behavior of valsartan in alkaline hydrolysis

Forced degradation in acidic media was performed by taking an aliquot of stock solution in a 10 ml volumetric flask and diluted up to the mark with HCl (0.75 N, 1.5 N) to obtain a final concentration of 100 mcg/ml. The flask was kept aside at room temperature for 2 h and neutralized. Appropriate aliquot was taken from the above solution and diluted with mobile phase to obtain a final concentration of 10 mcg/ml. Similarly, forced degradation in alkaline media was performed using 1.5 N NaOH. The representative chromatograms for acid and alkaline degradation studies are shown in Figures 2 and 3, respectively.

### Oxidative degradation

**Figure 4:**
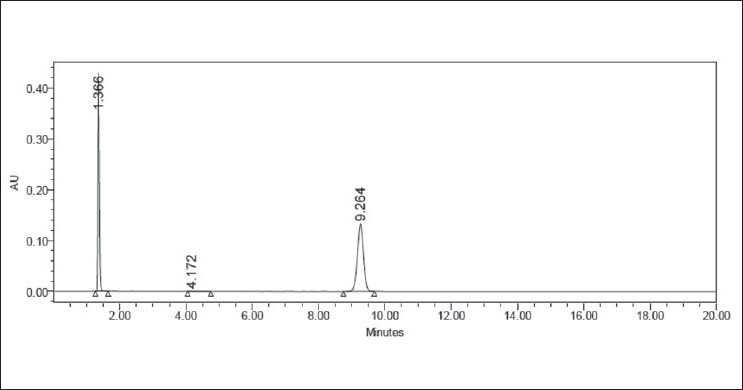
Typical chromatogram representing the degradation behavior of valsartan in oxidative hydrolysis

Oxidative degradation was performed by taking an aliquot of stock solution in a 10 ml volumetric flask and diluted up to the mark with hydrogen peroxide (3.0%, 10%) to obtain a final concentration of 100 mcg/ml. The flask was kept aside at room temperature for 2 h. Appropriate aliquot was taken from the above solution and diluted with mobile phase to obtain a final concentration of 10 mcg/ml. The representative chromatogram is shown in Figure 4.

### Photo stability

**Figure 5:**
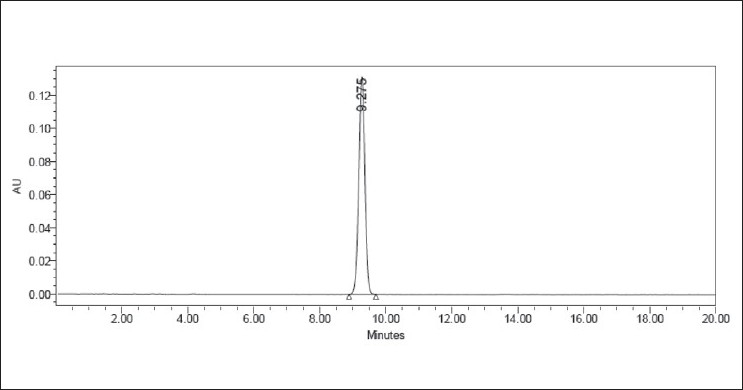
Typical chromatogram representing the degradation behavior of valsartan in liquid state photo-stability

Valsartan-API, tablet powder, and solutions of both were prepared and exposed to light to determine the effects of light irradiation on the stability of valsartan in solution and in the solid state. Approximately 50 mg of valsartan-API powder was spread on a glass dish in a layer that was less than 2 mm thickness and a solution of API (1 mg/ml) was prepared in mobile phase. Tablet powder was also prepared in the same way. All samples for photo-stability testing were placed in a light cabinet and exposed to light for 2 h resulting in an overall illumination of ≥210 Wh/m^2^ at 25°C with UV radiation at 320-400 nm. Control samples, which were protected with aluminum foil, were also placed in the light cabinet and exposed concurrently. Following removal from the light cabinet, all samples were prepared for analysis as previously described. The representative chromatogram is shown in Figure 5.

### Thermal stress studies

**Figure 6:**
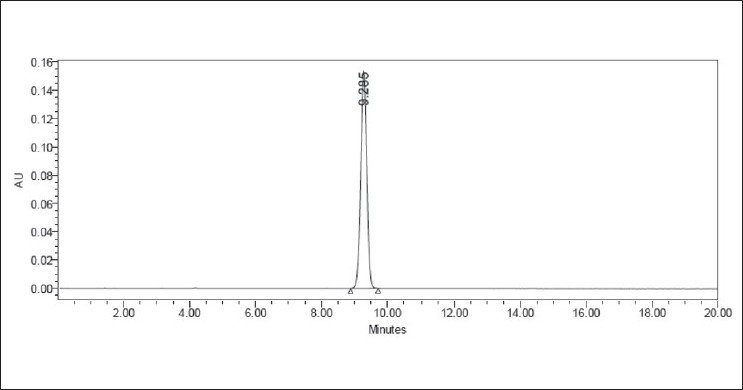
Typical chromatogram representing the degradation behavior of valsartan in temperature stress studies

For thermal stress, valsaratan-API, tablet powder, and solutions of both were prepared and exposed to a controlled temperature oven at 50°C and 80°C for 2 h. The representative chromatogram is shown in Figure 6.

## RESULTS AND DISCUSSION

### HPLC method: Development and optimization

The chromatographic column used was Waters Symmetry C_18_ 250 × 4.6 mm × 5 μm and it was maintained at ambient temperature for the separation and the method validated for the determination of valsartan tablets. The composition, pH, and the flow rate of the mobile phase were changed to optimize the separation conditions using stressed samples and the main related substances. A mobile phase consisting of 0.02 mM NaH_2_PO_4_ buffer (pH 2.5) and acetonitrile (58:42, %v/v) is selected at a flow rate of 1 ml/min, after several preliminary investigatory chromatographic runs. Under the described experimental conditions, all peaks were well defined and free from tailing. The effects of small deliberate changes in the mobile phase composition, pH, and flow rate were evaluated as a part of testing for method robustness.

### Results of forced degradation studies

Forced degradation studies were performed for bulk drug and tablet powder, to provide an indication of the stability indicating property and specificity of the proposed method. Intentional degradation was attempted to under different stress conditions to evaluate the ability of the proposed method to separate valsartan from its degradation products. Degradation was not observed in valsartan samples under stress conditions like acid hydrolysis, alkaline hydrolysis, and thermal exposure. However, mild degradation was observed when the drug was exposed to photolysis (UV cabinet at 320–400nm) and hydrogen peroxide. The concentration of valsartan was more slightly decreasing with time in hydrogen peroxide and UV light. This degradation is mainly observed in terms of loss of assay. Table 1 indicates the extent of degradation of valsartan under various stress conditions. Therefore, it may be concluded that valsartan is susceptible to degrade in oxidative and photolytic conditions. Photodiode array detection was used as an evidence of the specificity of the method and to evaluate the homogeneity of the drug peak. Chromatographic peak purity data were obtained from the spectral analysis report and a peak purity value greater than 990 indicates a homogenous peak. The peak purity values for analyte peaks in chromatograms of stressed samples were in the range of 999–1000 for drug substance, and in the range of 997–999 for tablets, indicating homogenous peaks and thus establishing the specificity of the assay method. Resolution between the analyte peaks and nearest peak was more than 2.0 in all the chromatograms. Figures 2 to 6 show the chromatograms of forced degraded samples. The degradation products were well resolved from valsartan, confirming the stability-indicating power of the method.

**Table 1:** Summary of forced degradation results

Stress condition	Time (h)	% Recovery	Retention time of the analyte	Peak purity
Acidhydrolysis (0.75 N HCl at RT	2	97.93	9.17	999
Acidhydrolysis (1.5 N HCl at RT)	2	96.46	9.24	999
Basehydrolysis (0.75N NaOH at RT)	2	97.13	9.16	999
Basehydrolysis (1.5N NaOH at RT)	2	97.13	9.16	999
Oxidation (3% H_2_O_2_ at RT)	2	96.33	9.16	999
Oxidation (10% H_2_O_2_ at RT)	2	37.40	9.26	999
Photolysis (UV cabinet at 320–400 nm)	2	94.41	9.27	999
Thermal treatment (50°C)	2	101.71	9.15	999
Thermal treatment (80°C)	2	101.42	9.28	999

RT-room temperature; API-Active Pharmaceutical Ingredient; cmean peak area is the average of three determinations

### Validation of the method

The analytical method was validated with respect to parameters such as linearity, limit of quantitation (LOQ), limit of detection (LOD), precision, accuracy, selectivity, recovery, and robustness/ruggedness.

### Linearity

Linearity was established by least squares linear regression analysis of the calibration curve. The constructed calibration curves were linear over the concentration range of 1–200 μg/ml. Peak areas of valsartan was plotted against their respective concentrations and linear regression analysis performed on the resultant curve. Correlation coefficient (*n*=3) was found to be more than 0.999 with %RSD values being less than 2% across the concentration ranges studied. Typically, the regression equation was

$$y\;=\;17905x\;+\;1137\;\left(R=0.9999\right)$$

### Limit of Detection and Limit of Quantitation

**Figure 7:**
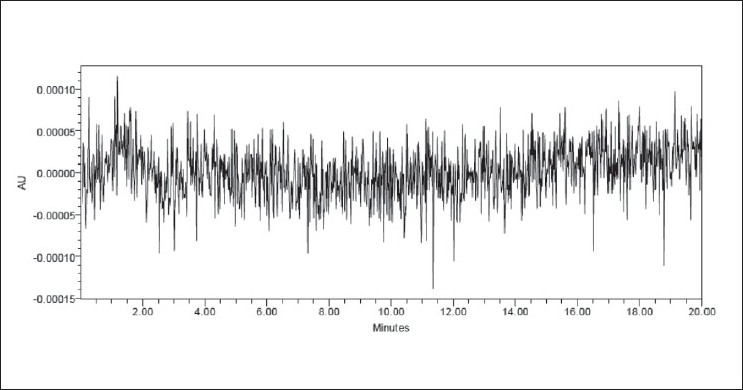
A representative chromatogram of the blank run

**Figure 8:**
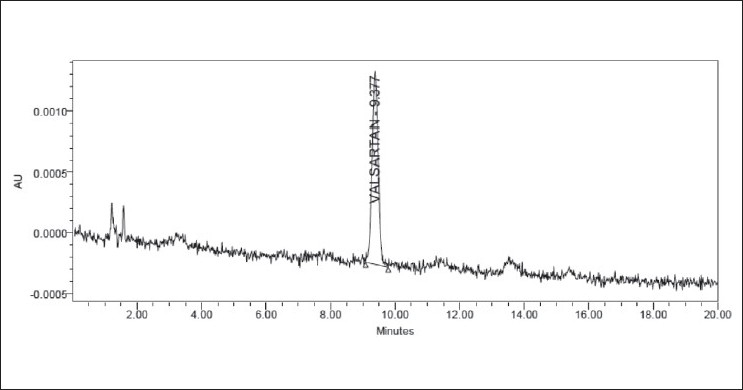
A representative chromatogram of valsartan at LOQ

The limit of quantitation (LOQ) of the present method was found to be 0.993 μg/ml with a resultant %RSD of 0.4% (*n* = 5). The limit of detection (LOD) was found to be 0.3 μg/ml. The representative chromatograms for blank run and chromatogram at LOQ were shown in Figures 7 and 8, respectively.

### Precision

Precision of the assay was investigated with respect to both repeatability and reproducibility. Repeatability was investigated by injecting nine replicate samples of each of the 25, 50, and 75 μg/ml standards where the mean concentrations were found to be 25.5, 50.2, and 74.8 μg/ml with associated %RSDs of 0.23, 1.24, and 0.42, respectively. Inter-day precision was assessed by injecting the same three concentrations over three consecutive days, resulting in mean concentrations of valsartan of 24.0, 50.3, and 74.1 μg/ml and associated %RSDs of 1.33, 1.59, and 1.7%, respectively. The ruggedness of the method was assessed by comparison of the intra- and inter-day assay results for valsartan undertaken by two analysts. The %RSD values for intra- and inter-day assays of valsartan in the cited formulations performed in the same laboratory by the two analysts did not exceed more than 2%, thus indicating the ruggedness of the method. The mean retention time of valsartan was 9.2 min with associated %RSD of 0.1%.

### Accuracy

The accuracy of the method was determined through the recovery test of the samples, using known amounts of valsartan reference standard. For the LC method, aliquots of 0.8, 1.0, and 1.2 ml of a valsartan standard solution (50 μg/ml) were added to three sample solutions containing a fixed amount of valsartan (50 μg) in diluent, respectively. Therefore, this recovery study was performed at a final concentration solution of 40.0, 50.0, and 60.0 μg/ml of valsartan. All solutions were prepared in triplicate and analyzed. Accuracy data for the assay following the determination of the compound of interest are summarized in Table 2.

**Table 2:** Accuracy data (n=3)

Amount of drug added (mg)	Mean amount of drug recovered (mg)	% drug recovery	% RSD
40	40.56±0.36	101.40	0.88
50	49.87±0.71	99.74	1.42
60	59.49±0.68	99.15	1.14

Data obtained from three replicates at each concentration.

### Specificity

The specificity of the LC method was evaluated to ensure that there was no interference from the excipients contained in pharmaceutical product or from products resulting from forced degradation. The results of stress testing studies in addition to that of monitoring standard solutions of the drug in the presence of their impurities indicated a high degree of specificity of this method. The degradation product(s) of the parent compound was found to be similar for both the tablets and API powder. All the degradation products formed during forced decomposition studies were well separated from the analyte peak, demonstrating that the developed method was specific and stability-indicating.

### Robustness Test

As recommended in the ICH Guidelines, a robustness assessment was performed during the development of the analytical procedure. The robustness of the method was investigated under a variety of conditions including changes of pH of the eluent, flow rate, and buffer composition. The degree of reproducibility of the results obtained as a result of small deliberate variations in the method parameters and by changing analytical operators have proven that the method is robust and the data are summarized in Table 3.

**Table 3:** Robustness testing of the method

Parameter	Modification	% Drug recovery
pH	2.4	100.1
2	2.5	100.3
3	2.6	99.8
Buffercomposition (mM)	0.01	98.5
	0.02	101.2
	0.03	100.8
Flowrate (ml/min)	0.9	100.5
	1.0	100.1
	1.1	101.3

Data obtained from three replicates at each concentration.

### System Suitability Parameters

System suitability parameters can be defined as tests to ensure that the method can generate results of acceptable accuracy and precision. The requirements for system suitability are usually developed after the completion of method development and validation. (Or) The USP (2000) defines parameters that can be used to determine system suitability prior to analysis. The system suitability parameters, such as Theoretical plates (N), Resolution (R), Tailing factor (T), were calculated and compared with the standard values to ascertain whether the proposed RP-HPLC method for the estimation of valsartan in pharmaceutical formulations was validated or not. The results are shown in Table 4.

**Table 4:** System suitability parameters

Parameter	Values obtained	Preferable values
Theoretical plates (N)	7848	>2500
Resolution (R)	4.8	>1.5
Tailing factor	1.07	<1.5

### Assay

**Figure 9:**
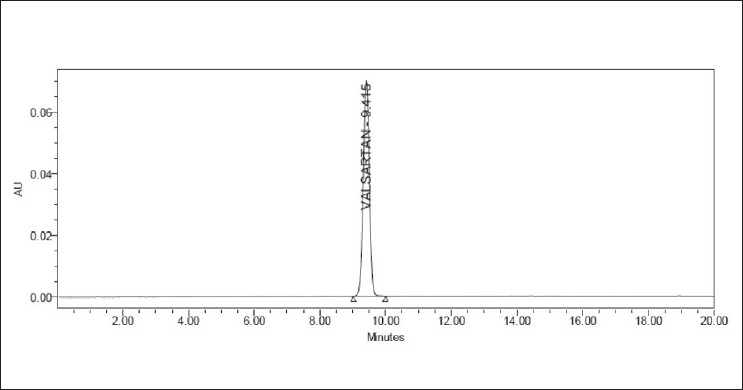
Typical chromatogram of valsartan in active pharmaceutical ingredient

**Figure 10:**
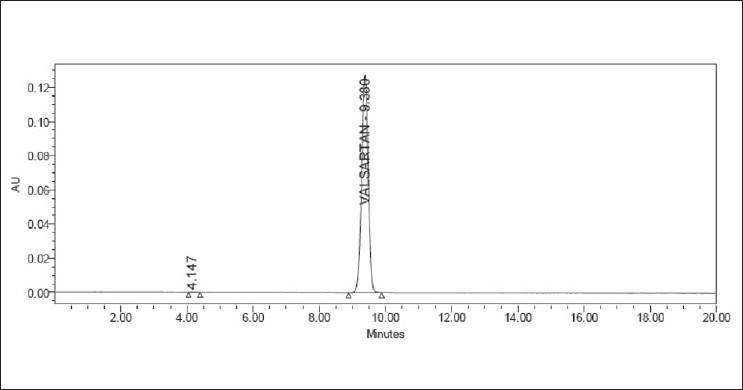
Typical chromatogram of valsartan in tablet dosage forms

The validated method was applied to the determination of valsartan in commercially available valzar tablets. Figures 9 and 10 illustrate two typical HPLC chromatograms obtained following the assay of valsartan reference standard solution and from valzar tablets, respectively. The results of the assay (*n* = 9) undertaken yielded 100.03% (%RSD = 1.40%) of label claim for valsartan. The observed concentration of valsartan was found to be 10.003±0.140 μg/ml (mean±SD). The mean retention time of valsartan was 9.38 min. The results of the assay indicate that the method is selective for the analysis of valsartan without interference from the excipients used to formulate and produce these tablets.

### Solution stability

The R.S.D. of assay of valsartan during solution stability experiments was within 1.0%. No significant change was observed in the content of valsartan during solution stability experiments. The experimental data confirmed that sample solutions used during assay and related substance determinations were stable up to 48 h.

## CONCLUSIONS

A simple, rapid, accurate, and precise stability-indicating HPLC analytical method has been developed and validated for the routine analysis of valsartan in API and tablet dosage forms. The results of stress testing reveal that the method is selective and stability-indicating. The proposed method has the ability to separate the drug from their degradation products, related substances, and excipients found in tablet dosage forms and can be applied to the analysis of samples obtained during accelerated stability experiments.
